# Unidirectional photodamage of pheophytin in photosynthesis

**DOI:** 10.3389/fpls.2013.00554

**Published:** 2014-01-13

**Authors:** Harvey J. M. Hou

**Affiliations:** Department of Physical Sciences, Alabama State UniversityMontgomery, AL, USA

**Keywords:** pheophytin, photosystem II, electron transfer, photodamage, photoinhibition

The primary reactions in photosynthesis take place in the reaction centers surrounded by light harvesting complexes (Blankenship, 2002; Diner and Rappaport, 2002; Frank and Brudvig, 2004). There are two types of photosynthetic reaction centers. The type I reaction center has iron-sulfur cluster as stable electron acceptor, such as photosystem I complexes, green bacterial and heliobacterial reaction centers, and the type II uses quinone as stable electron acceptor including photosystem II and purple bacterial reaction centers. The electron transfer in type II reaction center in unidirectional via the L-subunit of the reaction centers (Maroti et al., 1985; Hoerber et al., 1986; Martin et al., 1986; Michel-Beyerle et al., 1988). However, the electron transfer in the type I reaction center is different from that in the type II centers, which is bidirectionational (Guergova-Kuras et al., 2001; Li et al., 2006). The three dimensional structures of both types of reaction centers were determined at atomic resolution (Jordan et al., 2001; Ferreira et al., 2004; Loll et al., 2005; Amunts et al., 2007; Umena et al., 2011). The electron transfer pathways in both types of reaction centers are well established (Van Grondelle, 1985; Schatz et al., 1988; Fleming and van Grondelle, 1997; Dekker and Van Grondelle, 2000; Gobets and van Grondelle, 2001; Seibert and Wasielewski, 2003; Van Grondelle and Novoderezhkin, 2006). The primary electron donor in the reaction centers is a pair of chlorophyll molecules, and pheophytin is the primary electron acceptor (Klimov et al., 1977; Holzwarth et al., 2006).

Light is the only source of energy for photosynthesis; it can also be harmful to plants (Powles, 1984). During the recent 10 years the molecular processes of photoinhibition had been intensively studied (Adams and Demmig-Adams, 1993; Aro et al., 1993; Baker and Bowyer, 1994; Anderson et al., 1997; Asada, 1999; Melis, 1999; Niyogi, 1999; Adir et al., 2003; Telfer, 2005; Murata et al., 2007; Tyystjarvi, 2008; Kramer, 2010; Hou and Hou, 2013). The PSII complex is composed of more than 15 polypeptides and 200 pigment molecules. Because there are many pigment and protein molecules not related directly to the photoinhibition reaction, it is difficult to identify which molecule is photodamaged. The preparation of PS II reaction center D1/D2/cytochrome b-559 complex, which contains only a few polypeptides and pigments, can be a good material to meet the difficulty (Nanba and Satoh, 1989).

The PS II reaction center D1/D2/cytochrome b-559 complexes from higher plants contain five polypeptide subunits (Seibert et al., 2004). It contains six chlorophyll a (Chl a), two β-carotene (Telfer et al., 1987; Seibert et al., 1988; Gounaris et al., 1990; Kobayashi et al., 1990; Barbato et al., 1991). The PSII reaction center does not contain the quinone electron acceptors Q_A_ and Q_B_. It photochemical reaction is restricted to radical pair formation and recombination (Takahashi et al., 1987; Crystall et al., 1989; Wasielewski et al., 1989). Addition of exogenous electron donors and acceptors allows secondary electron flow reaction to occur. Therefore the D1/D2/cytochrome b-559 complex constitutes a good simple system for probing on the mechanisms from both acceptor-side and donor-side photoinhibition (Barber and Andersson, 1992; Barber and De Las Rivas, 1993; Yu et al., 1995). It has found that primary electron donor P_680_, accessary chlorophyll, carotene, amino acid residues such as histidine, and pheophytin are vulnerable to excess light.

The primary electron donor P_680_ of PSII can be damaged easily by exposure of strong light, when no additions are made (Telfer and Barber, 1989). Singlet oxygen is formed as a consequence of radical pair recombination (McTavish et al., 1989; Durrant et al., 1990). The generation of this highly toxic species causes initially a selective and irreversible bleaching of the chlorophylls that constitute P_680_ and a breakdown of the D1 protein to a 23-kDa fragment containing the N terminus of the complete protein (De Las Rivas et al., 1993). In the presence of a suitable electron acceptor such as silicomolybdate or decylplastoquinone, the P_680_^+^ lifetime is increased, and irreversible bleaching of β-carotene and chlorophyll are observed, which are independent of oxygen (Telfer et al., 1991). Under these conditions breakdown of the D1 protein leads to a 24-kDa fragment of C-terminal origin (Shipton and Barber, 1991).

The photobleaching at 680 nm is usually attributed to the photodamage of P_680_ in the PS II reaction center (Telfer et al., 1990). There is the significant overlapping of absorption of P_680_, accessary chlorophyll, and pheophytin in the PS II reaction center (Diner and Rappaport, 2002). Using high performance liquid chromatography (HPLC) the one pheophytin in PS II reaction center is photo damaged (Hou et al., 1995; Kuang et al., 1995). The time course of pheophytin photodamage showed the content of pheophytin decreases faster than that of chlorophyll, suggesting that light-induced damage of pheophytin and P_680_ occurred step by step in which pheophytin photodamage first and followed by P_680_ (Hou et al., 1996). The kinetics of the photodamage reaction of P_680_ suggested that the photodegradation product of P_680_ photodamage is possibly a pheophytin-like molecule (Peng et al., 1999).

A photoprotective hypothesis of pheophytin against photoinactivation induced from acceptor-side in PS II was proposed (Hou et al., 1996). When P_680_ bounding to D1 and D2 proteins is excited by light energy, primary charge separation takes place and the radial pair P_680_^+^ Pheo^−^ is formed, in which the excited state P_680_ ejects and transfers one electron to the primary electron acceptor pheophytin bound to D1 protein. Since primary quinone electron acceptor Q_A_ is lost in the preparation of PS II reaction center complex, the radical pair may be recombined and the P_680_ triplet is formed. The singlet oxygen generated by the reaction of the triplet P_680_ and oxygen in the solution is a highly toxic species to PS II reaction center. The pheophytin molecule probably bound to D2 protein firstly damaged by the singlet state oxygen. More excitation energy accumulated in PS II may cause the photodestruction of P_680_ and accessary chlorophyll. The photodamage of P_680_ and accessory chlorophyll resulted in the loss of photochemical activity of PS II. The role of the pheophytin molecule bound to D2 subunit is probably to remove the singlet state oxygen as a result protecting P_680_ against the photoinduced damage. A study of photoinhibition *in vitro* and *in vivo* concluded that photoinhibition in intact pea leaves at low temperature is mainly due to the acceptor-side mechanism (Shipton and Barber, 1994). The unidirectional photodamage and photoprotection of pheophytin is probably the important scheme by which the photoinhibitory reaction takes place in green plants *in vivo*.

As we discussed early, the role of the second or inactive electron transfer branch is unclear. Because the pheophytin molecule on D2 protein is the key component of the second electron-transfer branch in the PS II reaction center, the unidirectional photodamage of pheophytin infer that the role of the second branch is to protect the first one. In other words, the function of the second electron-transfer branch is to remove the singlet state oxygen species using the pheophytin molecule on D2 protein.

As PS II and purple bacterial reaction centers are both belong to the type-II reaction centers, the significant similarity in both structure and function are expected. During the early stages of the photoinhibition of isolated spinach PS II reaction center there is a loss of pheophytin (Hou et al., 1995; Kuang et al., 1995). To see if the process of photoinhibition is similar or different between the purple bacterial and plant reaction centers, the photosynthetic pigments and their correlation to the photochemical activity of the isolated reaction centers from *Rb. sphaeroides* were assessed (Hou et al., 2005). The experimental data demonstrated: (1) One bacteriopheophytin molecule associated with photochemical activity of the isolated reaction center from *Rb. sphaeroides* is damaged under strong light illumination; (2) The damaged bacteriopheophytin is likely located in the L subunit of the reaction center; (3) The special pair P_870_ undergoes a two-step photodamage reaction.

Further investigation on the photodamage mechanisms of bacteriopheophytin in the reaction centers from *Rb. sphaeroides* is conducted by using liquid chromatography-mass spectrometry (Hou, 2008). The experimental results show that one of the two bacteriopheophytin molecules in the purple bacterial centers accompanying with the complete loss of photoactivity is damaged under strong illumination due to the destruction of its chemical structure. Three degradation products of the bacteriopheophytin photodamage reactions are identified and probably produced by the hydration, de-hydrogen, and de-methylation of bacteriopheophytin molecule under strong light conditions. The main degradation product is probably formed by the de-methylation at C-8 in the bacteriopheophytin molecule.

**Figure 1:**
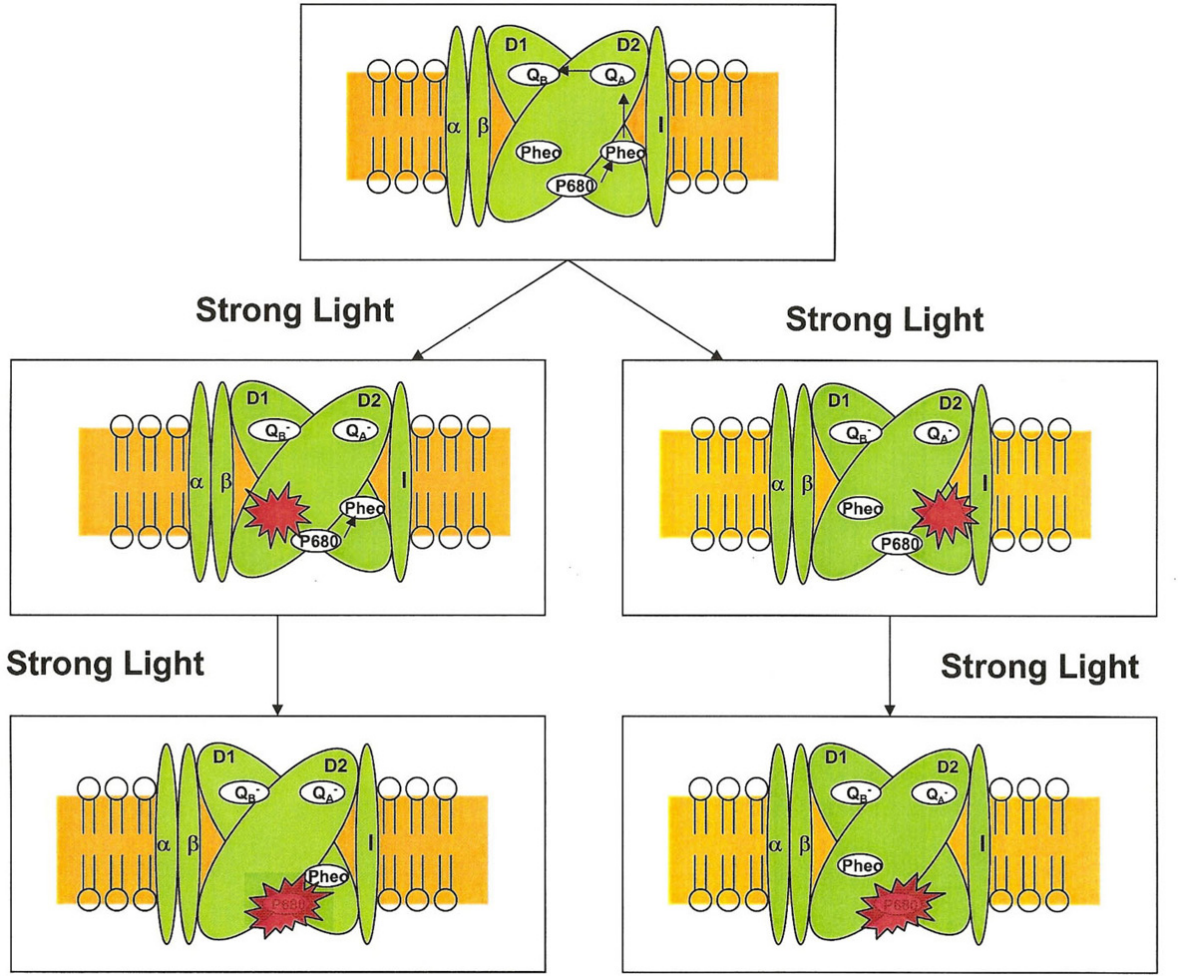
**Unidirectional photodamage of pheophytin in PS II reaction centers**.

Which pheophytin in PS II is more sensitive to strong light is currently unknown and need further investigation. The pigment analysis using HPLC and UV-vis-NIR spectroscopy indicates that one pheophytin in PS II and purple bacterial reaction center is more photosensitive than the other (Hou et al., 1996, 2005). The damaged pheophytin may be bound to D1 or D2 proteins (Figure 1). The HPLC and photochemical activity analysis suggested that the damaged pheophytin is likely in the inactive branch in PS II reaction center complex. However, we cannot exclude that possibility of the alternative option, i.e., the damaged pheophytin is located in the active is via D1 protein. This notion is supported by the observation that the photodamage of bacteriopheophytin in purple bacterial reaction center seems to be located in L-subunit.

The pheophytin mutants of *Synechocystis* sp. PCC 6803 might be useful to provide additional evidence on the site of the photosensitive pheophytin in photosynthesis. Two mutants, D1-Leu210His and D2-Leu209His, wherein the pheophytin in D1 and D2 protein is replaced by a chlorophyll, respectively. In these two mutants, there is only one pheophytin per reaction center. In the D1-Leu210His mutant, the only pheophytin in the reaction center is bound to D2 protein. If the photo damaged pheophytin in D2 protein, the photodamage of pheophytin would be expected in the D1-Leu210His mutant. Alternatively, if the pheophytin in D1 protein is more photosensitive, one would observe that photodamage of pheophytin in the D2-Leu209His mutant.

Unidirectionality of pheophytin photodamage might involve electron transfer reaction. For example, upon excitation with blue light (390 nm) a transient B-side charge separated electron transfer was observed in picoseconds at room temperature (Haffa et al., 2003). A series of mutations involving the introduction of potentially negative amino acids in the vicinity of P_870_ were characterized in terms of the nature of this reaction. B-side electron transfer in bacterial reaction center from *Rb. sphaeroides* was proposed to be possible photoprotection via rapidly quenching higher excited states of the reaction center (Lin et al., 2001).

Unidirectional electron transfer and photodamage are also discovered in other cofactors such as carotenoid in PS II. The reaction centers of PS II contain two types of β-carotene with different orientations. The β-carotene (I) absorbing at 470 and 505 nm is roughly parallel to the membrane plane, and β-carotene (II) absorbing at 460 and 490 nm seems to be perpendicular orientation (Breton and Kato, 1987) The linear dichroism signal at 485 nm in the PS II core complex CP47/D1/D2/cytochrome b-559 is decreased upon strong illumination (Hou et al., 2000). The observation may be explained as the conformation changes of β-carotene (II) pool, which tends to more perpendicular orientation respective to the membrane plane.

Multiple photoprotective hypotheses have been established including Mn-mediated UV photoinactivation (Hakala et al., 2005; Ohnishi et al., 2005; Wei et al., 2011; Hou et al., 2013), cytochrome b-559 cyclic electron flow or cytochrome b-559 reversible interconvertion between the two redox forms (Thompson and Brudvig, 1988; Barber and De Las Rivas, 1993; Shinopoulos and Brudvig, 2012), and a β-carotene photooxidation (Telfer et al., 2003; Alric, 2005; Shinopoulos et al., 2014). The unidirectional photodamage of pheophytin in photosynthesis is discovered. However the site of the damage pheophytin is unknown. The possible function of the pheophytin in photosynthesis is complex and likely involves forward electron transfer, photoprotection, structural support, photosynthetic evolution, and alternative electron transfer. Further investigation using biophysical techniques and mutagenic methods is required to approve or exclude these possibilities.
